# Targeted ferroptosis of myofibroblasts by tubeimoside I attenuates hypertrophic scar formation

**DOI:** 10.3389/fphar.2025.1654108

**Published:** 2025-09-29

**Authors:** Jianzhang Wang, Chen Wang, Yulei Jia, Fengchao Chen, Youbin Wang, Juan Du

**Affiliations:** ^1^ Department of Plastic and Cosmetic Surgery, Beijing Friendship Hospital, Capital Medical University, Beijing, China; ^2^ Department of Oncology, The Second Affiliated Hospital of Soochow University, Suzhou, China; ^3^ Department of Dermatology, Xuanwu Hospital, Capital Medical University, Beijing, China

**Keywords:** hypertrophic scar, fibroblasts, ferroptosis, PI3K/Akt signaling pathway, tubeimoside I

## Abstract

**Background:**

Hypertrophic scar (HS), the skin fibroproliferative disease, occurs after burn injury, traumatic injury, and surgery, resulting in high medical and economic burdens. Tubeimoside-I (TBMS1), a triterpenoid saponin monomer obtained from the Chinese medicinal herb Tubeimu, has demonstrated therapeutic potential in various diseases. In the present study, we explored the therapeutic effect of TBMS1 in the progression of HS.

**Methods:**

*In vitro* studies tested the effects of TBMS1 on the biological behaviors of hypertrophic scar fibroblasts (HSFs) were investigated by cell counting kit-8, flow cytometry, wound healing, transwell, and collagen gel contraction assays. Further, the regulatory mechanism of TBMS1 in alleviating HS was elucidated. *In vivo* experiments were utilized to reveal the influences of TBMS1 on HS formation.

**Results:**

*In vitro* studies indicated that TBMS1 hindered HSFs proliferation, migration, and myofibroblast activation. The PI3K/AKT signaling pathway mediates TBMS1-induced ferroptosis, which was accompanied by altered expression of NRF2, SLC40A1, and GPX4, ultimately suppressing cell proliferation and collagen synthesis. *In vivo* experiments confirmed that local TBMS1 injection exerted potent antifibrotic effects.

**Conclusion:**

This study revealed the effect of TBMS1 in activating ferroptosis, suggesting that inducing ferroptosis is probably a novel therapeutic strategy for hypertrophic scar.

## Introduction

Hypertrophic scar (HS), the pathological fibrotic disease, results from aberrant wound healing processes and manifests as excessive inflammation, hyperactivation of fibroblasts, and dysregulated synthesis and degradation of extracellular matrix components (particularly type I/III collagen) (Gauglitz et al., 2011). HS occurs in 40%–70% of patients with deep skin injuries such as burns, surgical wounds, or infections. These scars not only cause persistent pruritus, pain, and functional impairments, such as restricted joint mobility, but also lead to significant psychological and social adaptation challenges. Clinical management relies primarily on comprehensive approaches, including intralesional corticosteroid injections, pressure therapy, surgical excision, and laser therapy (Anderson et al., 2021). However, these treatments face limitations such as prolonged duration, high recurrence rates, and considerable interindividual variability in efficacy (Kivi et al., 2024; Rimmer et al., 2023; Khan et al., 2020), particularly due to the lack of specific targeted therapy addressing key pathogenic pathways. This has driven researchers to investigate novel interventions, including the modulation of cell death.

Dr. Brent R. Stockwell from Columbia University first proposed ferroptosis as a new iron-dependent programmed cell death pattern, unlike autophagy, apoptosis, or necrosis (Dixon et al., 2012). Ferroptosis can be triggered by phospholipid peroxidation, a process determined by reactive oxygen species (ROS), iron accumulation, and polyunsaturated fatty acid (PUFA)-containing phospholipids. Morphologically, ferroptosis leads to the rupture of the plasma membrane and cytoplasmic swelling, whereas ultrastructural observations reveal mitochondrial abnormalities. Both intracellular and intercellular signaling events, as well as environmental stress, can influence ferroptosis by regulating ROS levels and cell metabolism (Tang et al., 2021). Ferroptosis is tightly associated with pathologies of diverse disorders. For example, artesunate (ART) treatment significantly reduces liver injury and inhibits fibrotic scar formation in mouse models of liver fibrosis, with hepatocytes exhibiting morphological features of ferroptosis (Liu et al., 2024). Ferroptosis can also regulate the development of various diseases, including acute myeloid leukemia (AML), psoriasis, and hemolytic diseases (Jiang et al., 2021).

Natural compounds are important resources for medical development. Tubeimoside I (TBMS1), the triterpenoid saponin monomer obtained from the Chinese herbal medicine Tubeimu, is commonly used to treat inflammation. TBMS1 also has therapeutic effects on various diseases. For example, TBMS1 inhibits tumor progression by inducing cell apoptosis in human prostate (Yang et al., 2016), lung (Hao et al., 2015), liver (Yin et al., 2011), cervical (Xu et al., 2011), melanoma (Du J et al., 2020), and gastric (Zhang et al., 2013) cancers. Additionally, TBMS1 participates in multiple biological processes, including suppressing cell invasion (Bian et al., 2015), angiogenesis (Gu et al., 2016), and autophagy (Du J et al., 2020), as well as the induction of cell cycle arrest (Xu et al., 2013; Chen et al., 2012). However, its effect on regulating ferroptosis is unknown. We found that TBMS1 activated ferroptosis to mitigate skin fibrosis by inhibiting PI3K/AKT signaling pathway, demonstrating that TBMS1 is promising as an anti-HS therapeutic agent.

## Materials and methods

### Animal model

The Ethics Committee of Beijing Friendship Hospital, Capital Medical University approved the animal experiments (approval number: NO. 2025022010424352), which were implemented following the Guide for the Care and Use of Laboratory Animals. The animals were treated following the Committee on Publication Ethics guidelines. New Zealand white rabbits (3.0–4.0 kg) were intravenously injected with 1% pentobarbital sodium (10 g/L, 40 mg/kg) for anesthesia. An electric shaver was used for hair removal on the ventral side of each rabbit, followed by hair removal with a depilatory cream. A 1-cm biopsy punch was utilized for making 1-cm full-thickness circular wounds on the ventral side (n = 4 wounds/ear). The perichondrium from every wound bed was comprehensively eliminated out of the cartilage with the surgical blade. One week later, wounds were again exposed by removing crusts. When TBMS1 was injected, wounds were subjected to a subcutaneous injection of TBMS1 containing 40 μM DMSO (100 μL) or 400 μM DMSO (100 μL) on day 14 after wounding, whereas wounds from vehicle group received subcutaneous injection of 100 μL of DMSO at intervals of 7 days for 28 days. After 42 days, scar tissues were obtained from each group for H&E staining, Masson staining, or qRT-PCR.

### Cell culture

The culture medium included 10% fetal bovine serum (BI, Israel) as well as 1% penicillin-streptomycin, and the cells were cultured under 5% CO_2_ at 37 °C. For separating NSFs from HSFs, the skin tissues were rinsed thrice using phosphate-buffered saline (PBS) which contained 1% penicillin-streptomycin, while the subcutaneous adipose tissue was discarded through trimming. After cutting into pieces, the residual tissues were added to the culture medium. Both cell lines at passages 3–5 were harvested for subsequent analysis.

### Cell viability assay

Cell Counting Kit-8 (CCK-8, InCELLGene, United States) was used for measuring cell viability as recommended. Briefly, cells (4,000/well) were inoculated in a 96-well plate 48 h before the specified treatment. Next, every well was poured fresh medium (100 mL) containing CCK-8 reagent (10 mL) to replace the original medium. The absorbance was measured at 450 nm after 1 h of cell culture.

### Cell migration assay

Following cell inoculation in six-well plates, a pipette tip was used to generate linear defects after reaching 90% confluence. After 2 h of incubation with a solution containing 1 μg/mL mitomycin C, cells were rinsed by PBS thrice. At the same time, photographs of defect areas were obtained immediately, as well as 12 and 24 h post-wounding in each group. The results were examined using the ImageJ software (Bethesda, MD, United States).

### Transwell assay

We used 24-well inserts (8 µm thick; Corning, NY) to conduct the Transwell assay. Subsequently, bottom chamber was poured the 10% FBS-containing DMEM (500 μL), and upper chamber was poured FBS-free DMEM containing 5000 cells (200 µL). After 48 h, cells migrating to membranes were immersed prior to crystal violet staining. Images were captured with a microscope to count the stained cells.

### Collagen gel contraction assay

After the cells were seeded in 24-well plates containing a collagen suspension (500 μL, Cell Biolabs, San Diego, CA), the collagen gel was polymerized, and the gel was released from the plates through slight layering. Collagen gel areas were determined at 48 h.

### Apoptosis analysis

We used the Annexin V-FITC Apoptosis Detection Kit (BioLegend, San Diego, CA, United States) for detecting cell apoptosis following relevant instructions. After seeding into six-well plates, cells received corresponding treatment, trypsin digestion, and then transferred to 1.5-mL centrifuge tubes. Next, binding buffer (100 μL) was added to each tube, prior to 15 min of Annexin V-FITC (5 μL) and propidium iodide (PI, 10 μL) incubation away from light under ambient temperature. Finally, the cells were explored via flow cytometry (BD Biosciences, Franklin Lakes, NJ).

### Cell cycle analysis

The Cell Cycle and Apoptosis Analysis Kit (UElady, Suzhou, China) was utilized for detecting cell cycle. Following seeding into six-well plates, cells underwent corresponding treatment and trypsin digestion before being resuspended in PBS, followed by immersion in 75% ethanol at −20°C for 24 h. When centrifugation was performed to remove ethanol, the cells were incubated with PI-containing cell cycle staining solution away from light under ambient temperature for a 30-min duration. Then, cells were analyzed through flow cytometry (BD Biosciences, Franklin Lakes, NJ).

### Immunofluorescence analysis

After attachment, the cells were immersed for 15 min in a 4% formaldehyde buffer solution at ambient temperature. Permeability increased after 10 min of treatment with Triton X-100 (0.1%) in PBS. Next, the cells were incubated for 30 min with goat serum at room temperature and then with primary antibodies (α-SMA, 1:1000; 67735-1-Ig, 14395-1-AP; Proteintech) overnight. Later, cells underwent secondary antibody incubation (Alexa Fluor 488 and 594, Invitrogen) before 4′,6-diamidino-2-phenylindole (DAPI) nuclear staining.

### Western blotting

We used RIPA lysis buffer containing protease inhibitors for cell homogenization. A BCA protein assay kit (Beyotime, Shanghai, China) was applied in quantifying protein content. Through sodium dodecyl sulfate-polyacrylamide gel electrophoresis (Beyotime, Shanghai, China) for protein separation, proteins were transferred to polyvinylidene difluoride membranes (Millipore, Billerica, United States). Membranes were blocked using 5% nonfat milk at ambient temperature, before overnight primary antibody incubation under 4°C. Thereafter, membranes were subsequently rinsed by TBST thrice before 60 min of enzyme-conjugated secondary antibody incubation at ambient temperature. All signals were detected using ECL Western blot Substrate. The following antibodies (Proteintech) were used in this assay: PI3K (1:1000, 20584-1-AP), AKT (1:1000, 10176-2-AP), p-AKT (1:1000, 66444-1-Ig), COL Ⅲ (1:1000, 22734-1-AP), COL I (1:1000, 14695-1-AP), α-SMA (1:1000, 14395-1-AP), MMP2 (1:1000, 10373-2-AP), MMP9 (1:1000, 10375-2-AP), NRF2 (1:1000, 16396-1-AP), SLC40A1 (1:1000, 26601-1-Ig), GPX4 (1:1000, 30388-1-Ig), and GAPDH (1:5000, 60004-1-Ig).

### ROS measurement

After inoculation in six-well plates, the cells were subjected to different treatments before resuspension in serum-free medium containing 10 μM DCFH-DA (S0033S; Beyotime Biotechnology, China) and additional 20 min of incubation under 37°C. Fluorescence intensities were measured with a BD LSRFortessa flow cytometer (BD Biosciences, Franklin Lakes, NJ) via flow cytometry.

### Malondialdehyde (MDA) assay

The MDA content in the HSF lysates was examined using the MDA assay kit (A003-1–2; Nanjing Jiancheng, Nanjing, China) as recommended. When cells were homogenized, the MDA could react with thiobarbituric acid (TBA) in an acidic environment at 95°C to form the MDA-TBA adduct. The product absorbance was read with the microplate reader (Model 680, Bio-Rad, Hercules, CA, United States) at 532 nm.

### Fe^2+^ and Fe^3+^ assay

After inoculation (1.2 × 10^6^/well) in six-well plates, the cells were subjected to relevant treatments for 48 h. Intracellular Fe^2+^ contents were analyzed with the ferrous iron colorimetric assay kit (E-BC-K881-M, Elabscience, Wuhan, China). Intracellular Fe^2+^ contents and Fe^3+^ contents were analyzed with the total iron colorimetric assay kit (E-BC-K880-M, Elabscience, Wuhan, China). The absorbance was measured at 593 nm via spectrophotometry. The ratio of Fe^2+^/Fe^3+^ was evaluated as suggested.

### GSH and GSSG measurements

We used GSH and GSSG Assay Kits (S0053, Beyotime Biotechnology, China) to detect GSH and GSSG levels following specific instructions. After inoculation in six-well plates for the indicated treatments, the cells were suspended in protein removal agent, followed by two cycles of liquid nitrogen freezing and thawing at 37°C. Following 10 min of lysate centrifugation at 10,000 × *g*, supernatants were harvested. The absorbance was determined at 412 nm with the Model 680 Microplate Reader (Bio-Rad Laboratories). The ratio of GSH/GSSG was evaluated as suggested.

### Transient and stable transfection

We conducted temporary transfection as the guidelines provided by the manufacturer using lipofectamine 3000 (L3000075, Invitrogen, Carlsbad, United States). Tsingke (Beijing, China) supplied all the siRNAs. The targeting sequences were described below: si-cXT-1: 5′- CCA​CCU​GUU​UCA​CUA​AUA​ATT -3′.

### Transcriptome sequencing and analysis

Total cellular RNA was separated through the TRIzol reagent kit (Invitrogen, Carlsbad, CA, United States), with oligo (dT) beads being utilized to enrich total RNA. Next, fragmentation buffer was added to segment mRNA in short fragments, which were then synthesized into cDNA by random primers via reverse transcription. A QiaQuick PCR extraction kit (Qiagen, Venlo, Netherlands) was adopted for purifying cDNA fragments, which were later end-repaired and poly(A) inserted before ligation onto Illumina sequencing adapters. To select the ligation product size, agarose gel electrophoresis was performed before PCR amplification, and the products were sequenced on an Illumina NovaSeq 6000 instrument (Gene *Denovo* Biotechnology Co., Guangzhou, China).

### Quantitative real-time PCR (qRT-PCR)

We used an RNA Simple Total RNA Kit (Invitrogen) for separating total RNA. Next, cDNA was prepared with PrimeScript RT Master Mix (Yeasen, China). The target gene levels were subsequently examined via qRT-PCR by SYBR Premix EX Taq (Takara, China). The ^ΔΔ^CT strategy was used for measuring relative gene mRNA levels, and GAPDH was used as an endogenous reference. The results are presented as the fold change versus the control group. The following primers were used: TGF-β1: Forward (F) 5′- AGG​ACG​CCA​ACT​TCT​GCC​T-3′ and Reverse (R) 5′- AGG​ACC​TTG​CTG​TAC​TGG​GTG​T-3′; COL III: F 5′- TTC​CTT​TTG​TTC​TAA​TCT​TGT​CA-3′ and R 5′- TAG​CAC​CAT​TGA​GAC​ATT​TTG​A-3′; COL I: F 5′- TGA​GCC​AGC​AGA​TTG​AGA​AC-3′ and R 5′- CCA​GTG​TCC​ATG​TCG​CAG​A-3′; and α-SMA: F 5′- TTC​TGC​ATA​CGG​TCA​GCA​AT –3′ and R 5′- TTC​TGC​ATA​CGG​TCA​GCA​AT –3′.

### Reagents and drugs

TBMS1 (MCE, HY-N0890), ferrostatin-1 (MCE, HY-100579), necrosulfonamide (MCE, HY-100573), 740Y-P (MCE, HY-P0175), Z-VAD-FMK (MCE, HY-16658B), Erastin (MCE, HY-15763) and ML385 (MCE, HY-100523) were used.

### Statistical analysis

GraphPad Prism 9.0 for Windows was employed for all statistical analyses. Data are indicated as the mean ± standard deviation (SD). Between-group differences were analyzed through unpaired two-tailed Student’s t-tests, whereas among-group counterparts through one-way ANOVA (for a single independent variable) with Tukey’s multiple comparison test. Nonlinear regression was performed for analyzing IC_50_ values. The frequency of biological replicates is provided in the figure legends; *, **, and *** stand for p < 0.05, 0.01, and 0.001, respectively.

## Results

### TBMS1 inhibited HSF proliferation

**FIGURE 1:**
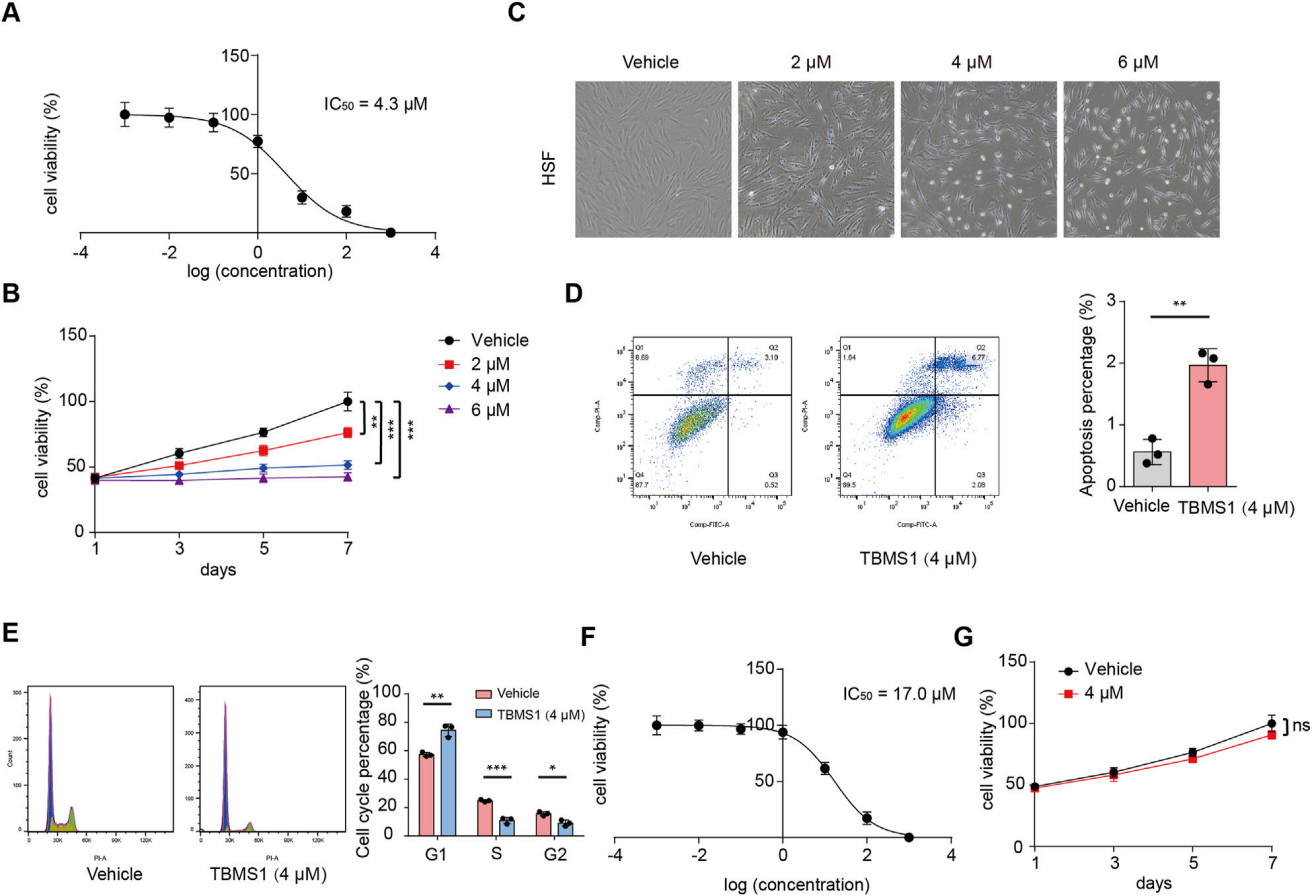
TBMS1 inhibited the proliferation of HSFs. **(A)** IC_50_ of TBMS1 for suppressing the proliferation of HSFs. **(B)** HSF viability following vehicle or TBMS1 treatment. **(C)** Cell morphology of HSFs following 48 h of treatment with vehicle or TBMS1 at various doses. **(D)** Flow cytometry and cell apoptosis analyses of HSFs treated with vehicle or TBMS1 (n = 3 separate assays). **(E)** Flow cytometry and cell cycle analyses of HSFs following treatment with vehicle or TBMS1 (n = 3 separate assays). **(F)** IC_50_ of TBMS1 for suppressing NSF proliferation. **(G)** NSF viability was assessed following vehicle or TBMS1 treatment (4 μM) (n = 3 separate assays). The data suggest mean ± SD. Assay **(B)** was examined by one-way ANOVA with Tukey’s multiple comparison test, whereas assays **(D)**, **(E)**, and **(F)** were examined through unpaired two-tailed Student’s t tests; *P < 0.05, **P < 0.01, and ***P < 0.001.

To explore the effect induced by TBMS1 on HSFs, HSFs received 48 h of TBMS1 treatment at varying doses. We carried out the CCK-8 assay for determining the half maximal inhibitory concentration (IC_50_) of TBMS1 on HSFs, which was 4.3 μM (Figure 1A). We selected 4 μM to be the indicated dose in later analysis. Based on CCK-8 assay, HSF viability after TBMS1 treatment was suppressed dose-dependently relative to vehicle group (Figure 1B). As observed via microscopy, after TBMS1 treatment, HSFs exhibited considerably altered morphology and reduced cell number (Figure 1C). Additionally, from flow cytometry, TBMS1 triggered HSF apoptosis while inducing the arrest of the HSF cell cycle (Figures 1D,E). To confirm the therapeutic efficacy of TBMS1 in primary non-fibrotic cells, we exposed normal skin fibroblasts (NSFs) to different doses of TBMS1 for 48 h. The IC_50_ value of TBMS1 for NSFs was 17.0 μM at 48 h, which was significantly higher than the value for HSFs (Figure 1F). Moreover, 4 μM TBMS1 did not significantly affect the viability of NSFs (Figure 1G). Therefore, TBMS1 inhibits HSF proliferation *in vitro* and is biosafe, making it a potential drug for treating HS.

### TBMS1 inhibited HSF migration

**FIGURE 2:**
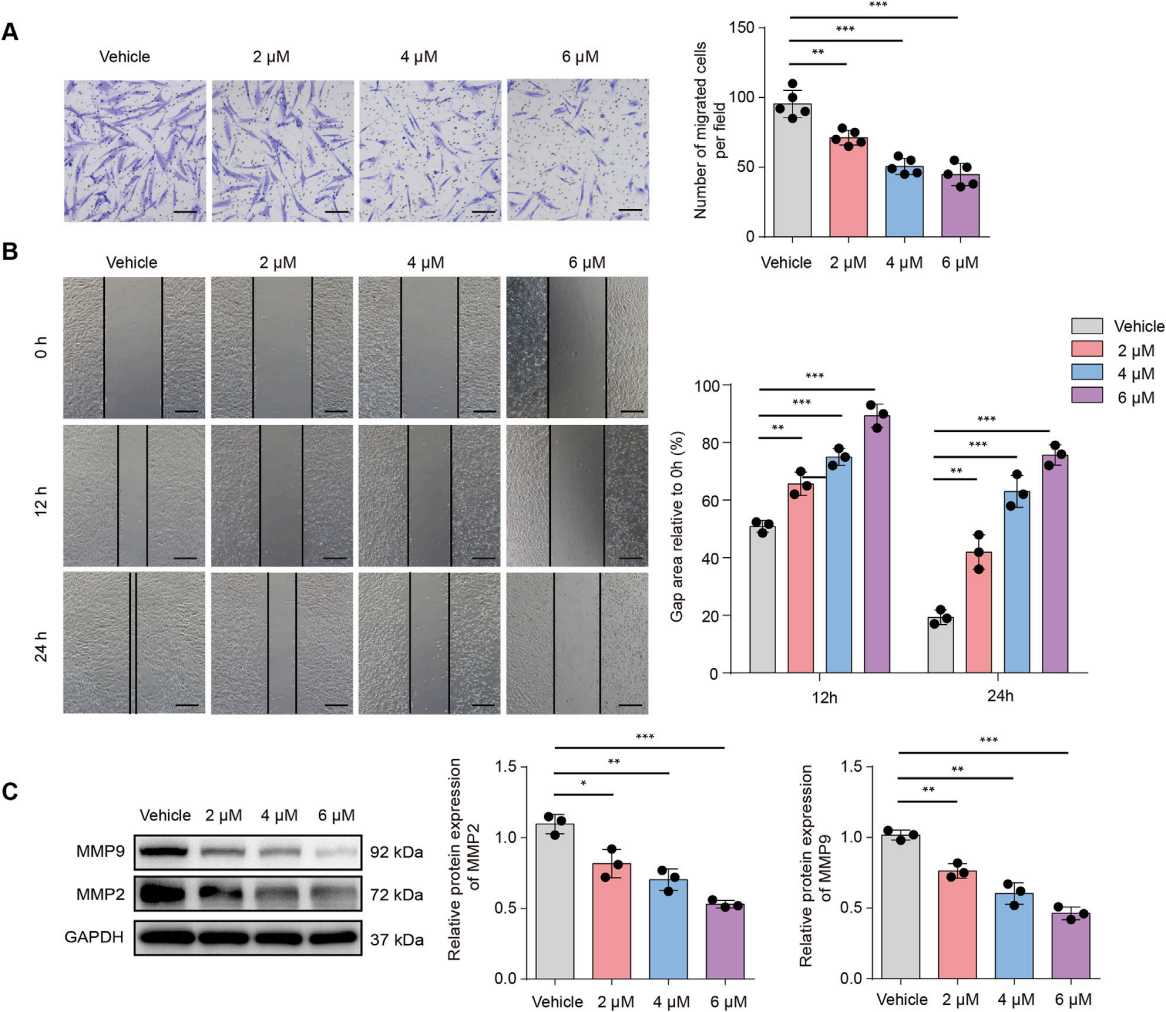
TBMS1 inhibited the migration of HSFs. **(A)** Images and Transwell assay quantitative analyses of the vehicle and TBMS1 groups (n = 5 separate assays); scale bar = 100 µm. **(B)** Images and wound healing quantitative analyses of the vehicle and TBMS1 groups (n = 3 separate assays). The scratch areas are represented by dotted lines; scale bar = 200 µm. **(C)** Western blotting was used for determining the MMP9 and MMP2 levels in vehicle-treated or TBMS1-treated HSFs. The data suggest mean ± SD. Experiments **(A)**, **(B)**, and **(C)** were examined through one-way ANOVA with Tukey’s multiple comparison test; *P < 0.05, **P < 0.01, and ***P < 0.001.

In HS, the activation and migration of fibroblasts into wound sites are important processes. This study conducted Transwell assays for assessing the efficacy induced by TBMS1 in HSF vertical migration. Our findings revealed that TBMS1 decreased HSF vertical migration (Figure 2A). We assessed the effect of TBMS1 inhibition on the horizontal migration capacity of HSFs by conducting a wound healing test. TBMS1-treated HSFs exhibited slower wound healing than vehicle-treated HSFs (Figure 2B). Additionally, TBMS1 reduced migration-related protein levels, like MMP2 and MMP9, in HSFs (Figure 2C).

### TBMS1 inhibited fibroblast activation and collagen expression

**FIGURE 3:**
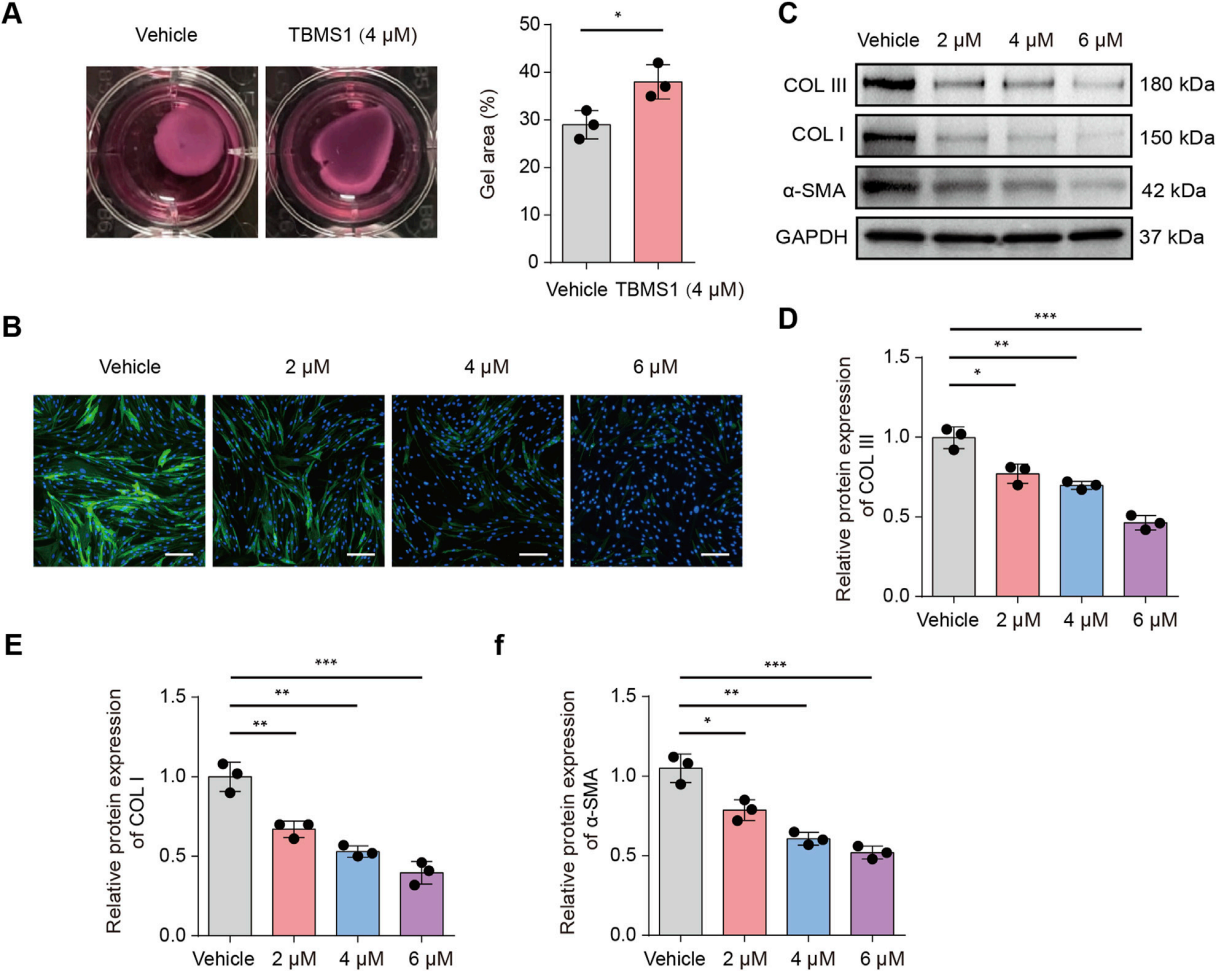
TBMS1 restrained HSF fibrotic phenotypes. **(A)** Representative images and collagen gel contraction quantitative analyses for the vehicle and TBMS1 groups (n = 3 separate assays). **(B)** Immunofluorescence analysis revealed α-SMA staining in vehicle-treated or TBMS1-treated HSFs; scale bar = 100 µm. **(C–F)** Western blotting was performed for determining COL I, COL III, and α-SMA contents of HSFs treated with vehicle or TBMS1. The data suggest mean ± SD. Experimental data **(C–F)** were examined via one-way ANOVA with Tukey’s multiple comparison test, whereas experimental data **(A)** were analyzed via an unpaired two-tailed Student’s t-test; *P < 0.05, **P < 0.01, and ***P < 0.001.

The therapeutic efficacy of TBMS1 in HSF cell contraction was subsequently examined by conducting a collagen gel contraction assay. TBMS1 decreased HSF-induced collagen gel contraction (Figure 3A). According to immunofluorescence analysis, α-SMA level decreased in the TBMS1-treated HSFs (Figure 3B). As revealed by Western blotting assays, TBMS1 downregulated fibrosis-associated proteins, like COL I, COL III, and α-SMA, in HSFs (Figures 3C-E).

### TBMS1 induced HSF ferroptosis

**FIGURE 4:**
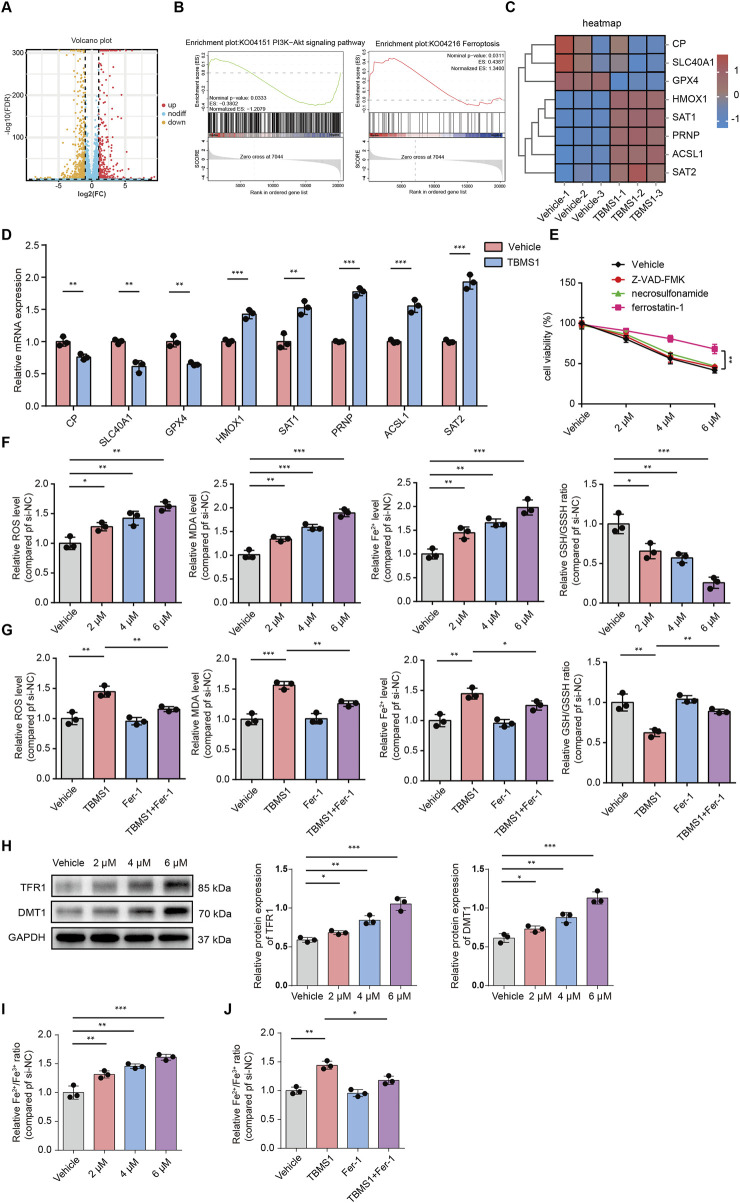
TBMS1 caused HSF ferroptosis. **(A)** The volcano plot displays significantly differentially-expressed genes (DEGs) in TBMS1-treated HSFs. **(B)** GSEA of DEGs in TBMS1-treated HSFs. Pathways related to PI3K/AKT pathway and ferroptosis are presented. **(C)** The heatmap shows typical TBMS1-regulated ferroptosis genes in HSFs. **(D)** The expression of the mRNAs of DEGs associated with ferroptosis in HSFs treated with vehicle or TBMS1 was estimated by qRT-PCR. **(E)** HSFs were treated with TBMS1 or TBMS1 combined with 1 µM ferrostatin-1, 10 µM Z-VAD-FMK, or 1 μM necrosulfonamide for 48 h before the measurement of cell viability (n = 3 separate assays). **(F)** ROS and MDA levels, intracellular Fe^2+^ contents, and the GSH/GSSG ratio in vehicle-treated or TBMS1-treated HSFs (n = 3 separate assays). **(G)** ROS and MDA levels, intracellular Fe^2+^ contents, and GSH/GSSG ratio of HSFs after treatment with TBMS1 or TBMS1 combined with ferrostatin-1 (1 µM) (n = 3 separate assays). **(H)** Western blotting illustrated TFR1 and DMT1 levels within HSFs after vehicle or TBMS1 treatment. **(I)** the Fe^2+^/Fe^3+^ ratio in vehicle-treated or TBMS1-treated HSFs (n = 3 separate assays). **(J)** the Fe^2+^/Fe^3+^ ratio of HSFs after treatment with TBMS1 or TBMS1 combined with ferrostatin-1 (1 µM) (n = 3 separate assays). The data suggest mean ± SD. Experiments **(E–J)** were examined via one-way ANOVA based on Tukey’s multiple comparison test, whereas assay **(D)** was performed via an unpaired two-tailed Student’s t-test; *P < 0.05, **P < 0.01, and ***P < 0.001.

**FIGURE 5:**
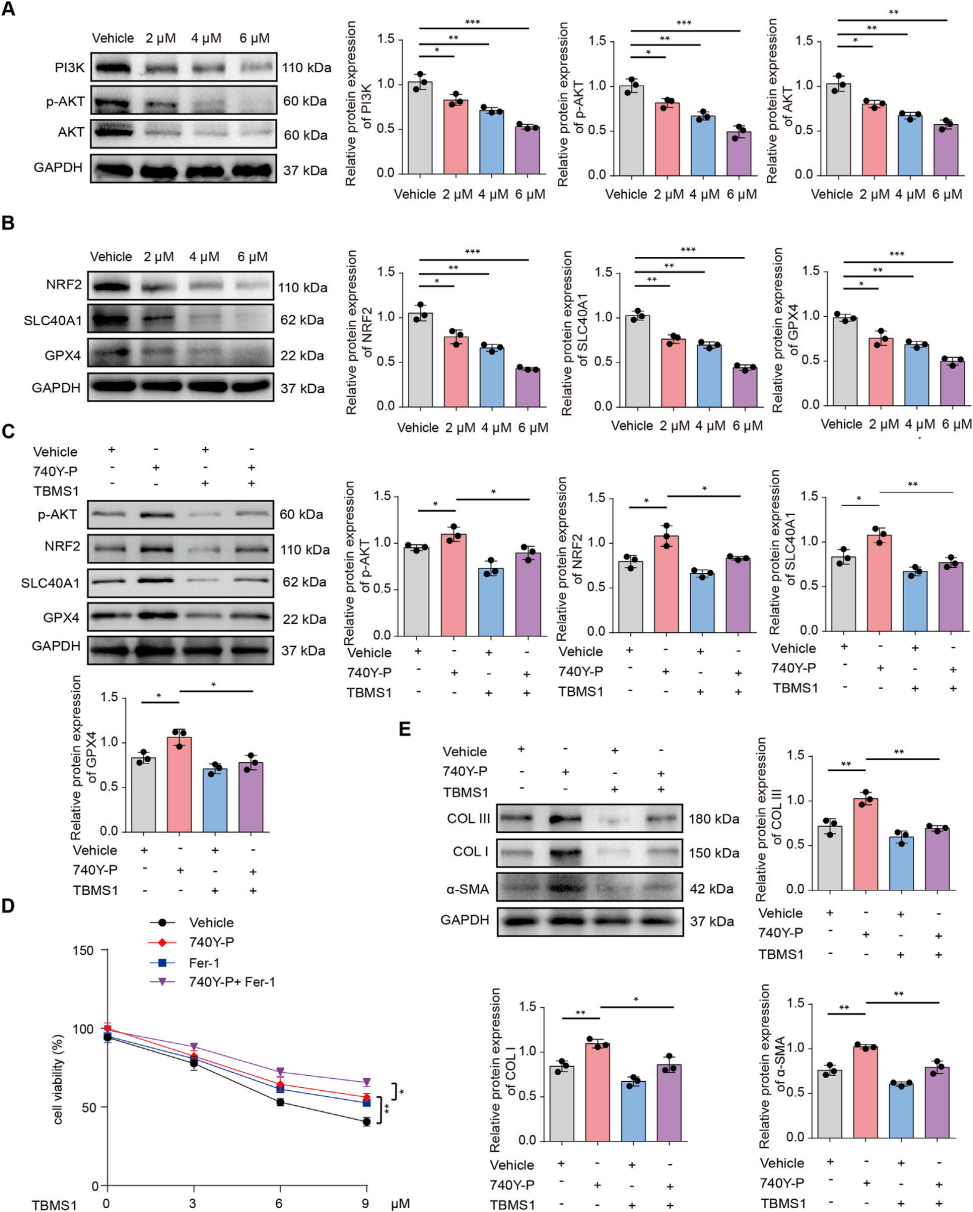
TBMS1 induced ferroptosis in HSFs by modulating PI3K/AKT pathway. **(A)** PI3K/AKT pathway levels in vehicle-treated or TBMS1-treated HSFs were detected by Western blotting. **(B)** Western blotting illustrated NRF2, SLC40A1, and GPX4 levels within HSFs after vehicle or TBMS1 treatment. **(C)** The p-AKT, NRF2, SLC40A1, and GPX4 protein levels in HSFs under TBMS1 or TBMS1 combined with 740Y-P (30 μM) treatment; 740Y-P is a PI3K/AKT pathway activator**. (D)** Cell viability under 48 h of treatment with TBMS1 or TBMS1 + 1 μM ferrostatin-1 (Fer-1) or 30 μM 740Y-P in HSFs (n = 3 separate assays). **(E)** COL I, COL III, and α-SMA proteins within HSFs treated with TBMS1 or TBMS1 combined with 740Y-P (30 μM). The data suggest mean ± SD. Assays **(A–E)** were examined based on one-way ANOVA with Tukey’s multiple comparison test; *P < 0.05, **P < 0.01, and ***P < 0.001.

We performed RNA-sequencing analysis to observe downstream molecular changes caused by TBMS1. Compared to those in the control group, 1253 DEGs (386 upregulated and 867 downregulated) in the TBMS1-treated group presented a greater than twofold change (Figure 4A). Additionally, upon gene set enrichment analysis (GSEA), differentially expressed molecules of TBMS1-treated versus control groups were enriched into ferroptosis pathways (Figure 4B). Some key genes closely related to ferroptosis were significantly regulated in the TBMS1-treated group, as revealed by RNA-sequencing analysis (Figure 4C). Next, we carried out qRT-PCR for assessing DEG contents, such as CP, SLC40A1, GPX4, HMOX1, SAT1, PRNP, ACSL1, and SAT2. TBMS1 administration considerably increased the levels of ferroptosis-associated genes, such as HMOX1, SAT1, PRNP, ACSL1, and SAT2, but decreased the levels of CP, SLC40A1, and GPX4, consistent with alterations in SLC40A1 and GPX4 protein levels (Figures 4D, 5B). Therefore, we inferred that TBMS1 triggers HSF ferroptosis.

We also found that TBMS1 induced the death of HSFs dose-dependently, but such effect could be abolished after ferrostatin-1 (the ferroptosis-specific inhibitor) rather than Z-VAD-FMK (the apoptosis inhibitor) and necrosulfonamide (the necroptosis inhibitor) treatment (Figure 4E). Then, we analyzed how TBMS1 affects ferroptosis in HSFs. First, flow cytometry assays were conducted to detect changes in ROS levels in HSFs. The results showed that TBMS1 promoted ROS production and that ferrostatin-1 abolished this effect (Figures 4F,G). Similarly, TBMS1 significantly increased the level of intracellular MDA, which was mostly reversed by ferrostatin-1. TBMS1 increased the ferrous ion (Fe^2+^) concentration in HSFs in a dose-dependent manner, whereas treatment with ferrostatin-1 decreased the concentration of Fe^2+^ (Figures 4F,G). In contrast, TBMS1 decreased the ratio of GSH/GSSH (a resistance indicator of ferroptosis of HSFs) dose-dependently (Figures 4F,G). We detected the expression of DMT1 and TfR1 by Western blot and the results showed that the TBMS1 stimulation enhanced the protein levels of DMT1 and TfR1 HSFs (Figure 4H). The concentration of the labile iron pool (LIP) in HSFs was a critical determinant of ferroptotic sensitivity. Therefore, we have also conducted experiments to explore dynamic interconversion between Fe^2+^ and Fe^3+^ under TBMS1 treatment in HSFs. The results showed that TBMS1 increased the ratio of Fe^2+^/Fe^3+^, whereas treatment with ferrostatin-1 decreased the ratio of Fe^2+^/Fe^3+^ (Figures 4I,J).

### TBMS1-induced ferroptosis is regulated via PI3K/AKT pathway

**FIGURE 6:**
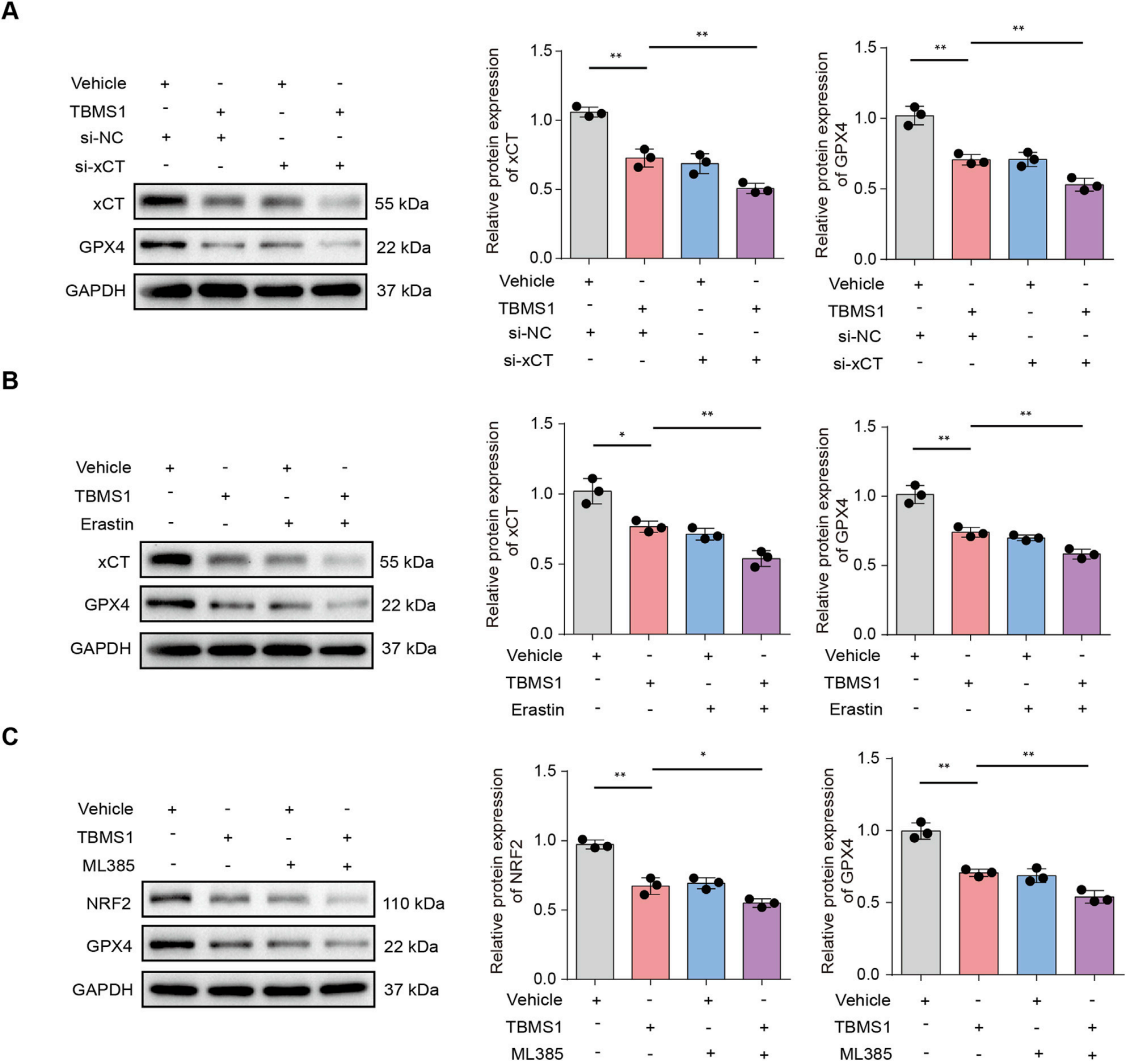
TBMS1 induced ferroptosis in HSFs by regulating NRF2/xCT/GPX4 pathway. **(A)** The x-CT, and GPX4 protein levels in HSFs under TBMS1 or TBMS1 combined with si-xCT treatment. **(B)** The x-CT, and GPX4 protein levels in HSFs under TBMS1 or TBMS1 combined with Erastin (5 μM) treatment. **(C)** The NRF2, and GPX4 protein levels in HSFs under TBMS1 or TBMS1 combined with ML385 (5 μM) treatment. The data suggest mean ± SD. Experiments **(A–C)** were examined via one-way ANOVA based on Tukey’s multiple comparison test; *P < 0.05, **P < 0.01, and ***P < 0.001.

GSEA was conducted to reveal differentially-expressed molecules in TBMS1-treated versus control groups enriched into PI3K/AKT pathway (Figure 4B). Other studies have suggested that activating phosphocreatine kinase 3 (PI3K) results in the phosphorylation of protein kinase B (AKT), thereby triggering nuclear factor erythroid 2-related factor 2 (NRF2) (Zheng et al., 2025; He et al., 2025; Pan et al., 2025). Immunoblotting analysis revealed that TBMS1 inhibited the PI3K/AKT signaling pathway (Figure 5A). NRF2 overexpression upregulated various antioxidant genes, such as SLC40A1 and GPX4, and these genes protected against ferroptosis (Wang J. et al., 2025). We also found that TBMS1 treatment markedly downregulated NRF2, SLC40A1, and GPX4 levels within HSFs dose-dependently (Figure 5B). The PI3K agonist 740Y-P was used to further investigate whether TBMS1 induces ferroptosis in HSFs through PI3K/AKT/NRF2 pathway. Besides, NRF2, SLC40A1, and GPX4 proteins were upregulated following 740Y-P treatment (Figure 5C). Further studies revealed that 740Y-P attenuated the anti-proliferative and ECM synthesis effects of TBMS1 on HSFs (Figures 5D,E). In order to further reveal the regulatory relationship between GPX4 and system xCT, we performed the following supplementary experiments and found that knocking down SlC7A11 using si- SLC7A11 significantly decreased the expression of GPX4 in HSFs (Figure 6A). Similarly, the SLC7A11 inhibitor Erastin also reduced on the expression of GPX4 in HSFs (Figure 6B). We employed ML385, a targeted NRF2 inhibitor, to investigate its effect on GPX4 in HSFs and found that ML385 significantly enhanced TBMS1-induced downregulation of GPX4 (Figure 6C).

### TBMS1 ameliorated HS in rabbits

**FIGURE 7:**
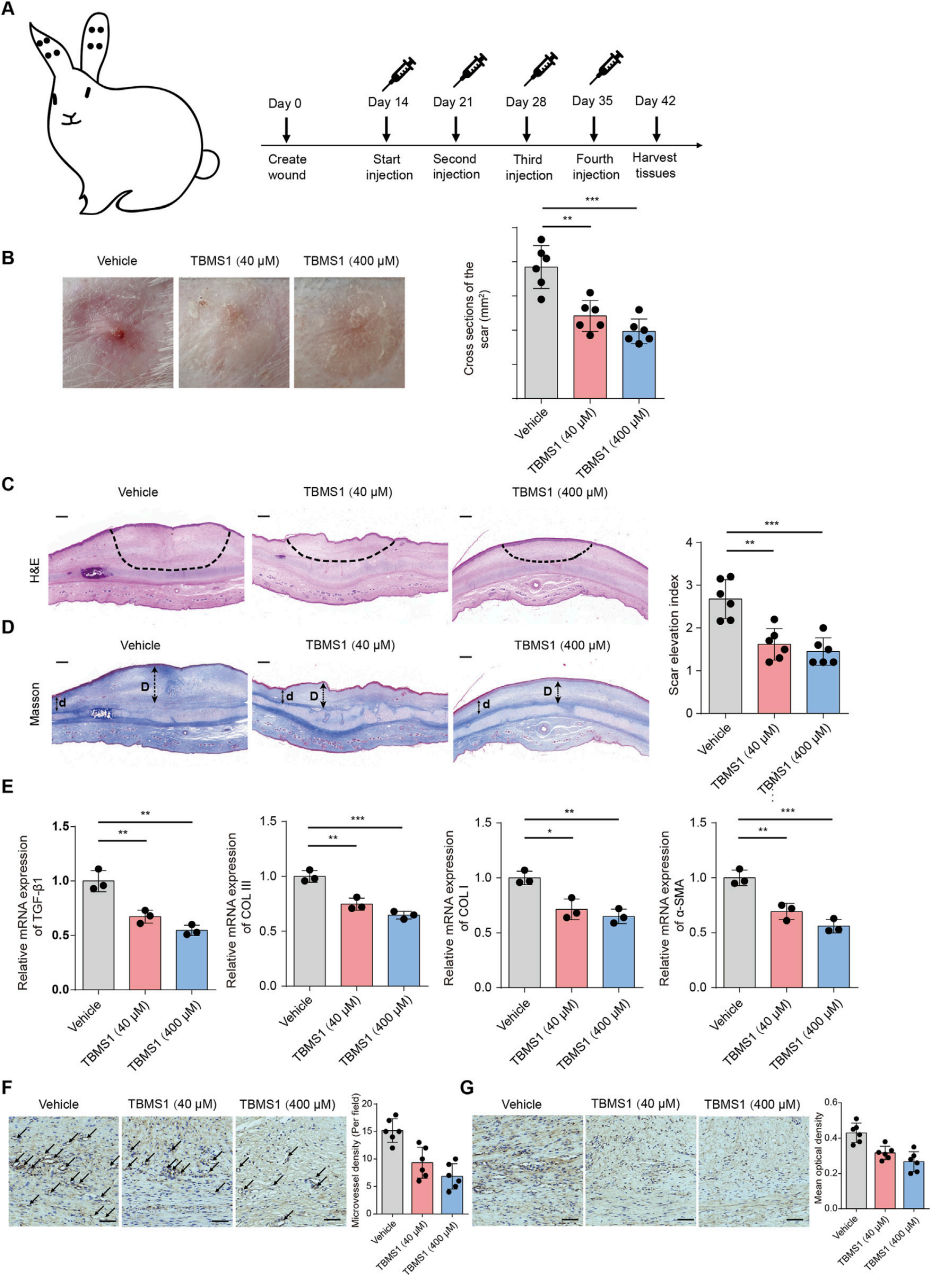
TBMS1 ameliorated rabbit ear HS formation. **(A)** The sketch map shows the rabbit ear HS model and TBMS1 treatments (40 and 400 μM). **(B)** Typical gross photographs show HS formation in rabbits. **(C)** Histological staining (H&E) and quantitative analysis of scar cross-sections of rabbit skin fibrotic tissues (n = 6 rabbits/group); scale bar = 200 μm. **(D)** Masson staining and quantitative analyses of rabbit scar SEIs (n = 6 rabbits/group). The long **(D)** and short (d) arrows represent the HS and neighboring unaffected skin tissue thicknesses, respectively. The “scar elevation index” was the D/d ratio; scale bar = 200 μm. **(E)** TGF-β1, COL I, COL III, and α-SMA mRNA expression within rabbit scar tissues following the administration of TBMS1 were measured via qRT-PCR (n = 3/group). **(F)** Representative images of immunohistochemical staining for CD31 in scar tissues from control group and TBMS1-treated group. Black arrows indicate blood vessels stained with an anti-CD31 antibody (n = 6 rabbits/group). scale bar = 100 μm. **(G)** The immunohistochemical staining for VEGF in scar tissues from control group and TBMS1-treated group (n = 6 rabbits/group). scale bar = 100 μm. The data suggest mean ± SD. Experiments **(C–G)** were assessed via one-way ANOVA with Tukey’s multiple comparison test; *P < 0.05, **P < 0.01, and ***P < 0.001.

To ascertain the *in vivo* efficacy of TBMS1, a rabbit model of ear HS was constructed (Figure 7A). Compared to the control (vehicle) group, TBMS1 treatment reduced hyperemia and increased scarring, whereas the scars in the TBMS1-treated groups were relatively soft and flat (Figure 7B). Histologically, TBMS1 administration reduced the scar tissue cross-sectional area (Figure 7C) and the scar elevation index (SEI) (Figure 7D). TGF-β1, COL I, COL III, and α-SMA exert important effects on HS formation. TBMS1 reduced the mRNA levels of these factors in TBMS1 rather than vehicle groups (Figure 7E). These *in vivo* findings conform to *in vitro* results and demonstrated that TBMS1 strongly inhibits skin fibrosis through the downregulation of ECM synthesis-related gene expression. Angiogenesis plays an important role in the early phase of hypertrophic scar formation. Therefore, to further investigate the ability of TBMS1 to influence the angiogenic processes during rabbit ear scar tissue formation, we performed a quantitative comparison of the density of CD31-positive vasculature in scar tissue sections in the control, TBMS1-treated groups. The immunohistochemical staining results revealed that less blood vessels per field in the TBMS1-treated group relative to the control group (Figure 7F). In addition, the expression of VEGF was significantly reduced in the TBMS1-treated group (Figure 7G).

## Discussion

This study identified the inhibition of TBMS1 against fibroblast proliferation and suggested that TBMS1 activated ferroptosis in fibroblasts. According to our findings, PI3K/AKT pathway has a key effect on regulating TBMS1-mediated ferroptosis. By conducting *in vivo* experiments, we demonstrated that TBMS1 effectively suppressed HS growth.

TBMS1 is a bioactive triterpenoid saponin with therapeutic potential across multiple diseases. It exerts antitumor effects by inducing apoptosis through caspase activation and BCL-2 inhibition (Yang et al., 2016) while also suppressing metastasis via the downregulation of MMP-2/9 (Cao et al., 2019). However, no study has reported the therapeutic efficacy of TBMS1 in HS. As scar cells share hyperproliferative characteristics with tumor cells, we designed experiments that not only confirmed the inhibitory effects of TBMS1 on scar growth but also elucidated the underlying molecular mechanisms. TBMS1 exhibited antifibrotic activity primarily by inhibiting fibroblast proliferation and promoting ferroptosis. *In vivo* experiments revealed that intralesional injection of TBMS1 effectively suppressed the growth of HS. Therefore, TBMS1 exerts a strong inhibitory effect on fibroblast proliferation.

Ferroptosis, a new type of cell death, has great potential in treating fibrotic diseases. Activated fibroblasts in fibrotic diseases are markedly susceptible to ferroptosis because of their high metabolic demands and iron accumulation characteristics (Horn and Tacke, 2024). Targeted modulation of key ferroptosis pathways, such as the inhibition of GPX4 activity in liver fibrosis or the application of iron chelators in idiopathic pulmonary fibrosis, can effectively induce fibroblast death and reduce collagen deposition (Wang et al., 2025a; Pei et al., 2022). Natural compounds such as ART promote ferroptosis by activating ACSL4-dependent lipid peroxidation, which also offers innovative therapeutic avenues for HS (Liu et al., 2024). Our study revealed that TBMS1 induced fibroblast death exclusively through ferroptosis, as indicated by the characteristic increase in ROS levels and corresponding biomarker alterations. After establishing the role of ferroptosis in TBMS1-mediated suppression of fibrosis, we investigated the underlying molecular pathways involved. The RNA-seq results revealed significant enrichment of altered pathways in the PI3K/AKT signaling axis in TBMS1-treated fibroblasts.

The PI3K/AKT signaling pathway can regulate ferroptosis susceptibility through the antioxidant system and iron metabolism. The PI3K/AKT signaling pathway regulates ferroptosis through the upregulation of NRF2, which is a key antioxidant stress regulator (Gao et al., 2025; Wang et al., 2025b; Yin et al., 2025). Moreover, activation of the pathway upregulated GPX4 (via mTORC1) and SLC7A11 (via NRF2), suppressing the accumulation of lipid ROS (Cheng et al., 2022; Fu et al., 2023). In contrast, PI3K/AKT inhibition (e.g., by LY294002) reduces GPX4 activity and promotes ACSL4-mediated lipid peroxidation (Zhang et al., 2025). In cancer therapy, targeting PI3K/AKT pathway-mediated ferroptosis can be the potential treatment (Sun et al., 2024). Thus, PI3K/AKT pathway serves as a central negative regulator of ferroptosis. We found that TBMS1 reduced PI3K, AKT, and p-AKT levels in fibroblasts in time-dependent and dose-dependent manners, while activating the PI3K/AKT pathway rescued ferroptosis-related protein levels. Further information on the signaling pathways and molecular mechanisms underlying the antifibrotic effects of TBMS1 may help develop new treatments against HS and offer resources for developing antifibrotic drugs.

Although we were the first to demonstrate that TBMS1 inhibits the growth of HS by inducing ferroptosis, this study had several limitations. For example, efficacy comparisons with conventional scar treatments were lacking. Additionally, since drugs are generally multitargeted, we have not yet determined whether TBMS1 induces other forms of cell death. Therefore, the therapeutic mechanisms related to TBMS1 in HS require further investigation. However, this work lays the theoretical foundation for applying TBMS1 in treating HS.

## Conclusion

**FIGURE 8:**
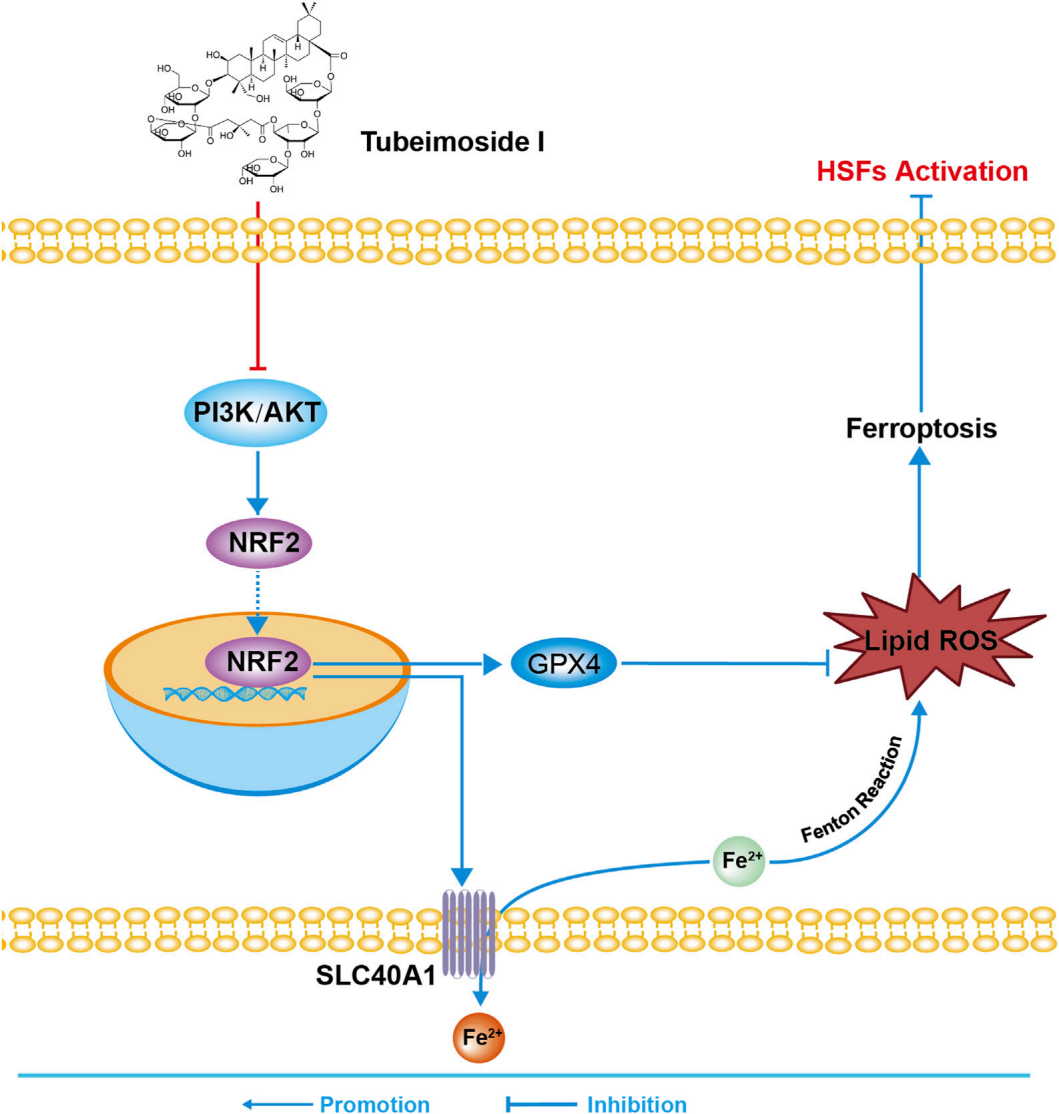
The sketch map shows the molecular mechanism of TBMS1 in treating HS.

To summarize, TBMS1 inhibited HS by triggering myofibroblast ferroptosis via the PI3K/AKT/NRF2-ferroptosis pathway (Figure 8). These results support the use of TBMS1 as a PI3K/AKT signaling pathway inhibitor and ferroptosis inducer, which may be translated to treat HS.

## Data Availability

All the citations and data included in this manuscript are available upon request by contact with the corresponding author. The original contributions presented in the study are publicly available. The RNA-seq data used in this study have been deposited in the National Center for Biotechnology Information's Sequence Read Archive (Sequence Read Archive study accession code PRJNA1282663) (https://www.ncbi.nlm.nih.gov/bioproject/PRJNA1282663).
